# Prevalence of geriatric depression in the Kavre district, Nepal: Findings from a cross sectional community survey

**DOI:** 10.1186/s12888-019-2258-5

**Published:** 2019-09-03

**Authors:** Kedar Manandhar, Ajay Risal, Oshin Shrestha, Nirmala Manandhar, Dipak Kunwar, Rajendra Koju, Are Holen

**Affiliations:** 1 Dhulikhel Hospital, Kathmandu University Hospital, Dhulikhel, Kavre Nepal; 2 Department of Community Medicine, Kathmandu University School of Medical Sciences, Dhulikhel, Kavre Nepal; 3 Department of Psychiatry, Kathmandu University School of Medical Sciences, GPO Box 11008, Dhulikhel, Kavre Nepal; 4 Sheer Memorial Hospital College of Nursing, Banepa, Kavre Nepal; 5 Department of Medicine, Kathmandu University School of Medical Sciences, Dhulikhel, Kavre Nepal; 6 Department of Mental Health, Norwegian University of Science and Technology, Trondheim, Norway

**Keywords:** Depression, Elderly, Lifestyle, Mental health, Prevalence

## Abstract

**Background:**

The increasing elderly population worldwide is likely to increase mental health problems such as geriatric depression, which has mostly been studied in high-income countries. Similar studies are scarce in low-and-middle-income-countries like Nepal.

**Methods:**

A cross-sectional, population-based, door-to-door survey was conducted in randomly selected rural and urban population clusters of the Kavre district, Nepal. Trained nurses (field interviewers) administered structured questionnaires that included a validated Nepali version of the Geriatric Depression Scale short form (GDS-15) for identifying geriatric depression among the elderly (≥60 years) participants (*N* = 460). Those scoring ≥6 on GDS-15 were considered depressed. Logistic regression analysis explored the associations of geriatric depression with regard to socio-demographic information, life style, family support and physical well-being.

**Results:**

Of the total 460 selected elderly participants, 439 (95.4%) took part in the study. More than half of them were females (54.2%). The mean age was 70.9 (± 8.6) years. Approximately half (50.6%) were rural inhabitants, the majority (86.1%) were illiterate, and about three-fifths (60.1%) were living with their spouses. The gender-and-age adjusted prevalence of geriatric depression was 53.1%. Geriatric depression was significantly associated with rural habitation (AOR 1.6), illiteracy (AOR 2.1), limited time provided by families (AOR 1.8), and exposure to verbal and/or physical abuse (AOR 2.6).

**Conclusion:**

Geriatric depression is highly prevalent in Kavre, Nepal. The findings call for urgent prioritization of delivery of elderly mental health care services in the country.

**Electronic supplementary material:**

The online version of this article (10.1186/s12888-019-2258-5) contains supplementary material, which is available to authorized users.

## Background

Worldwide, the elderly population is rapidly growing. The number of the elderly aged 60 years and above is projected to double by 2050; the biggest increments will be found in the low-and middle-income (LAMI) countries [1]. These demographic changes are expected to create substantial challenges for the social and health care services with a major increase in chronic diseases, physical disabilities, and mental health problems such as depression [1, 2].

Geriatric depression is an important public health concern that impairs the quality of life (QoL) and imposes financial costs on the family and society [3–8]. The Global Burden of Disease Study 2017 (GBD 2017) ranked depression as the third most leading cause of the years of life lost to disability (YLD) [9]. Most studies on geriatric depression have been carried out in high-income countries [2, 10, 11]; similar studies are relatively few in the LAMI countries [11] such as Nepal.

Nepal is one of the resource-poor countries within the South-East Asia Region (SEAR). About one quarter of the total population live below the international poverty line [12]. The population is approximately 30 millions; around one tenth is 60 years and above [13–15]. Though the percentage of the elderly in Nepal is not as high as in the high-income countries, there is, nevertheless, an increasing proportion of the elderly in the population, partly due to declining infant mortality and the crude birth rate [14].

The prevalence of elderly depression is expected to be high in Nepal, as per the results from other available studies [16–22]. However, most of the existing local studies have been conducted in geriatric homes [16–18, 21], or in clinical settings [19]. Very few studies are performed in community settings [20, 22, 23], and those have mostly been carried out in Kathmandu, the capital of Nepal [20, 22]. The prior findings are thus not likely to be representative of the entire geriatric population of the country with its vast socio-cultural and ethnic diversity.

Accordingly, we conducted a study of a community based sample, accommodating both the rural and urban populations. The Kavre district was a rather suitable candidate in this regard. The primary objective of the study was to estimate the prevalence of depression among the elderly in the Kavre district. The secondary objective was to identify factors associated with geriatric depression such as demographic, life-style, family related support and physical well-being. The purpose was to inform the health policy makers about the needs, and to provide foundations for better health-care resource allocations in the country.

## Methods

### Ethics

The Institutional Review Committee of Kathmandu University School of Medical Sciences (IRCKUSMS), Dhulikhel Hospital, Kavre, Nepal approved the study protocol.

All invited participants were informed about the nature, purpose and procedures of study. They were also informed that they were free to withdraw their consent and discontinue the research interviews at any time. In addition, they had the opportunity to ask any questions they would like regarding the study. Before the interviews began, written consent was given by those participants who could read and write, while fingerprints were collected from those who were unable to do that.

### Study design and sampling

Data collection was made in January and February 2019. The design was a cross-sectional, population-based, door-to-door survey based on field interviews conducted in the Kavre district, which falls under Province no 3, Nepal. It comprises seven rural and six urban municipalities. According to the Central Bureau of Statistics (CBS) 2011 [15], the total population was 381,937 (males 182,936 [47.9%] and females 199,001 [52.10%]. In this population, 36,912 were elderly aged 60 years or above (9.6% [males 9.7% and females 9.6%]).

### Inclusion and exclusion criteria

The inclusion criteria for age was 60 years and above. The participants should live in the Kavre district and be able to speak Nepali. Moreover, elderly persons who had lost the ability to speak or had major hearing loss, who were disoriented were excluded, i.e., they were unable to give correct answers to at least two of the three following questions (i) time of day [morning, day and evening], (ii) location [outside or inside the house], and (iii) weekday of the data collection [Sunday to Saturday], and those who were residing in old-age homes.

### Sample size and sampling procedures

We estimated a sample size of 460 persons by using the formula Z^2^pq/d^2^ assuming a prevalence of depression (p) 60.0%, a precision (d) 5.0% of p with 95% confidence interval (CI), and adding a 20% non-response rate.

We used a multistage systematic random sampling procedure to recruit eligible participants. In the first stage, four municipalities, two out of six urban and two out of seven rural municipalities of the Kavre district were selected by using a lottery method. We allocated equal proportions of participants (115 elderly) for each municipality to ensure a balanced representation from rural and urban areas. In the second stage, we selected at least 50% of the wards (administrative units) from each selected municipality also by using the lottery method.

The field interviewers sketched the ward map. They listed and numbered households with elderly inhabitants. This was achieved by help from the Female Community Health Volunteers (FCHVs); they are local women working voluntarily to enrich the outreach health care services at the community level. From the list, we selected households using a systematic random sampling technique. In the third stage, we selected one elderly person from each household. If there were two or more elderly in the household, one of them was selected randomly using the lottery method.

### Participants

Of the potential 460 selected elderly individuals, 13 declined to participate, six had impaired hearing, and two were unavailable. These 21 elderly (10 male, 11 female; age 60–85 years [13 urban and 8 rural]) were regarded as non-participants. Finally, 439 persons participated in the study. Thus, the proportion of participation was 95.4%.

A comparison of representativity has been made in Table 1 between the age and gender distribution of our selected samples of the elderly and the figures from the CBS [15]. The selected sample proved to be quite representative of the elderly population of the Kavre district, despite a rather modest overrepresentation of females and persons of age 70 years and above.

**Table 1:** Age and gender distribution of elderly in a randomly selected sample (*N* = 439) from the Kavre district, Nepal compared with national figures^a^

Age (in years)	All		Male		Female	
	Kavre^a^	Sample	Kavre^a^	Sample	Kavre^a^	Sample
	*n* (%)	*n* (%)	*n* (%)	*n* (%)	*n* (%)	*n* (%)
60–64	11,437 (30.9)	124 (28.2)	5425 (30.6)	56 (27.9)	6012 (28.6)	68 (28.6)
65–69	9336 (25.3)	77 (17.5)	4529 (25.6)	35 (17.4)	4807 (25.0)	42 (17.6)
70–74	6937 (18.8)	105 (24.0)	3355 (19.0)	45 (22.4)	3582 (18.6)	60 (25.2)
≥75	9202 (25.0)	133 (30.3)	4386 (24.8)	65 (32.3)	4816 (25.1)	68 (28.6)
Total	36,912 (100.0)	439 (100.0)	17,695 (47.9)	201 (45.8)	19,217 (52.1)	238 (54.2)

^a^National Population and Housing Census 2011 [15]

### Study instruments

Face-to-face interviews were done by trained nurses (field interviewers) using a structured questionnaire. The questionnaire was developed in English and translated into Nepali and tested in the field before initiating the data collection (Additional file 1). The final structured interview consisted of six parts (i) personal and demographic characteristics (age, gender, habitation, educational and marital status); (ii) life style related questions (alcohol consumption); (iii) family support (time provided by family, financial support, perceived respect from family, and verbal and/or physical abuse by family); (iv) questions that map the elderly person’s physical condition with regard to chronic physical health problems; the subjective report of any of the following diseases: diabetes, chronic obstructive pulmonary diseases [COPD], heart disease, cancer, paralyses and/or mobility problems; (v) questions from the Geriatric Depression Scale short form (GDS-15) [24]. A detailed description of the scale is given below. The last part (vi) consisted of biometric measurements such as height, weight, waist circumference and blood pressure (BP).

### Geriatric depression scale short form (GDS-15) - Nepali version

We used the Geriatric Depression Scale short form (GDS-15) to scan the potential presence of depressive symptoms [24]. The instrument had already been used in Nepal [22, 23] and in other Asian countries [25–27] including India [28] and Pakistan [29].

The original English version GDS-15 [24] was translated into Nepali. For the validation, the Nepali version was administered to the total of 106 participants from the Kavre district (mean age 68.1) by trained nurses. The participants were later blindly interviewed by the local consultant psychiatrists for possible geriatric depression according to the criteria of the International Classification of Diseases-10 (ICD-10).

In the validation study, 5/6 was found to be the optimal cut-off point also in Nepal. The internal consistency was found to be 0.79, and the sensitivity (Se) was 86.3% and the specificity (Sp) was 74.5%. Further details of the validation study will be published elsewhere (Risal et al.).

The GDS-15 consists of 15 items; they are focusing on the psychological symptoms that the person felt during the past week. Each item is rated in a yes/no format. Among them, 10 items (2, 3, 4, 6, 8. 9, 10, 12, 14 & 15) indicate the presence of depression when answered “yes” (positive), while the remaining 5 items (1, 5, 7, 11 & 13) indicated depression when answered “no” (negative). The potential total score ranged from 0 to 15 [24].

### Assessment of geriatric depression

A score of 5 or less was considered to be within the normal range and classified as a “no case” of geriatric depression, while 6 or more endorsements were considered to indicate a caseness of geriatric depression according to the validation study.

### Statistical analysis

We estimated the crude prevalence of geriatric depression with a 95% confidence interval (CI), and we adjusted the prevalence for gender and age according to the population distribution of the elderly aged ≥60 years of the Kavre district, as found in the data from the CBS [15].

The depression status of the GDS-15 was used as the dependent variable and the responses were dichotomized into yes (case of geriatric depression) or no (no case of geriatric depression).

Moreover, we dichotomized the socio-demographic variables like age (< 75 or ≥ 75 years), gender (male or female), habitation (urban or rural), educational status (literate or illiterate), and marital status (married or unmarried/widowhood/separate); life style factors including alcohol consumption (yes or no); family support in terms of the time provided by the family (yes or no), source of financial support (her/himself or family), perceived respect from family (yes or no), and verbal and/or physical abuse (no or yes); and any physical condition that included limited mobility (‘able to go out of home’ or ‘not able to go out of home’); and finally, any chronic physical health problem (yes or no).

Bivariate and multivariate logistic regression analyses with odds ratios [ORs] and adjusted ORs [AORs] respectively were used, each with 95% CIs to investigate the associations of geriatric depression with the above mentioned variables.

The *p*-values < 0.05 were considered statistically significant.

The statistical analyses were carried out using the Statistical Package for Social Science software (IBM SPSS Statistics 21, Chicago, USA).

## Results

### Socio-demographic characteristics

The socio-demographic characteristics of the participants have been presented in the Table 2. Of the 439 participants, 201 (45.8%) were males and the rest 238 (54.2%) were females. The mean age was 70.9 (SD 8.6) years. A large majority (86.1%) were illiterate, and about three-fifths (60.1%) were married and living with their spouse.

**Table 2:** Socio-demographic characteristics of a randomly drawn sample of the elderly (*n* = 439)

Variables	N	%
Age (in years)		
< 75	306	69.7
≥ 75	133	30.3
Gender		
Male	201	45.8
Female	238	54.2
Residence		
Urban	217	49.4
Rural	222	50.6
Educational status		
Literate	61	13.9
Illiterate	378	86.1
Marital status		
Married	264	60.1
Unmarried/widowed/divorce	175	39.9
Current smoker		
No	321	73.1
Yes	118	26.9
Current alcohol consumer		
No	331	75.4
Yes	108	24.6

### Prevalence

The crude prevalence of geriatric depression was 56.0% (95% CI 51.3–60.7). The gender-and-age adjusted prevalence was 53.1% and the age-adjusted female-to-male ratio was 1.14.

The prevalence of geriatric depression was found to be higher among females than among males (59.2% vs. 52.2%), more prevalent among those above 75 years (66.9% vs. 51.3%) and also, among those from rural areas compared to those from urban areas (63.5% vs. 48.4%). In addition, geriatric depression was more prevalent among the illiterate (59.8% vs. 32.8%) and among those who were unmarried, widowed or divorced (63.3% vs. 49.2%) (Table 3).

**Table 3:** Prevalence of geriatric depression by socio-demographic characteristics of elderly (*n* = 439)

Variables	n	Prevalence n (%)	95% CI
Age (in years)			
< 75	306	157 (51.3)	45.6–57.0
≥ 75	133	89 (66.9)	58.2–74.8
Gender			
Male	201	105 (52.2)	45.1–59.3
Female	238	141 (59.2)	52.7–65.5
Residence			
Urban	217	105 (48.4)	41.6–55.2
Rural	222	141 (63.5)	56.8–69.9
Educational status			
Literate	61	20 (32.8)	21.3–46.0
Illiterate	378	226 (59.8)	54.7–64.8
Marital status			
Married	264	130 (49.2)	43.1–55.4
Unmarried/widowed/divorce	175	116 (63.3)	58.8–73.2

*CI* confidence interval

### Socio-demographic associations

The associations between geriatric depression and the socio-demographic variables have been presented in Table 4.

**Table 4:** Bivariate and multivariate logistic regression analyses of associations of geriatric depression with dichotomized variables of socio-demographic information, life style, family support and physical well-being among the elderly (*n* = 439)

Variables	*n* (%)	Bivariate analyses		Multivariate analyses	
		OR (95% CI)	*p*	AOR^a^ (95% CI)	*p*
Socio-demographic variables					
Age (in year)					
< 75	306 (69.7)	reference	–	reference	–
≥ 75	133 (30.3)	1.9 (1.3–2.9)	0.003	1.3 (0.8–2.1)	0.36
Gender					
Male	201 (45.8)	reference	–	reference	–
Female	238 (55.2)	1.3 (0.9–1.9)	0.14	0.9 (0.5–1.4)	0.53
Residence					
Urban	217 (49.4)	reference	–	reference	–
Rural	222 (50.6)	1.9 (1.3–2.7)	0.001	1.6 (1.1–2.4)	**0.046**
Educational status					
Literate	61 (13.9)	reference	–	reference	–
Illiterate	378 (86.1)	3.1 (1.7–5.1)	< 0.001	2.1 (1.1–4.0)	**0.037**
Marital status					
Married	265 (60.1)	reference	–	reference	–
Unmarried/widowhood/separate	175 (39.1)	2.0 (1.4–3.0)	< 0.001	1.4 (0.9–2.4)	0.16
Current alcohol consumption					
No	331 (75.4)	reference	–	reference	–
Yes	108 (24.6)	1.5 (0.9–2.4)	0.059	1.6 (0.9–2.7)	0.065
Family support variables					
Time given by family					
Yes	274 (62.4)	reference	–	reference	–
No	165 (37.6)	3.0 (2.0–4.5)	< 0.001	1.8 (1.1–2.9)	**0.012**
Financial support					
Self-manage	277 (63.1)	reference	–	reference	–
Family support	162 (36.9)	0.8 (0.5–1.3)	0.18	0.8 (0.5–1.2)	0.38
Perceived respect from family					
Yes	389 (88.6)	reference	–	reference	–
No	50 (11.4)	4.1 (1.9–8.7)	< 0.001	2.1 (0.9–4.9)	0.069
Verbal and/or physical abuse by family					
No	348 (79.3)	reference	–	reference	–
Yes	91 (20.7)	3.8 (2.2–6.6)	< 0.001	2.6 (1.4–4.8)	**0.002**
Physical well-being					
Chronic physical health problems					
No	281 (64.0)	reference	–	reference	–
Yes	158 (36.0)	1.7 (1.1–2.5)	0.013	1.8 (1.2–2.8)	**0.012**
Mobility (ability to go out of home)					
Able to go out of home	291 (66.3)	reference	–	reference	–
Unable to go out of home	148 (33.7)	2.3 (1.5–3.4)	< 0.001	1.4 (0.9–2.3)	**0.046**

^a^adjusted age, gender, residence, education status, marital status, current alcohol consumption, time given by family, financial support, perceived respect from family, verbal and/or physical abuse by family, physical mobility and presence of physical chronic health problem; *AOR* Adjusted odds ratio, *CI* confidence interval

In the bivariate analyses, the geriatric depression was more frequent in the older age group (≥75 years) (OR 1.9 [95% CI1.3–2.9]; *p* = 0.003), among those residing in the rural areas (OR 1.9 [95% CI 1.3–2.7]; *p* = 0.001), among those who were illiterate (OR 3.1 [95% CI 1.7–5.1]; *p* < 0.001), and among those being unmarried/widowhood/divorced (OR 2.0 [95% CI 1.4–3.0]; *p* < 0.001).

In the multivariate analyses, however, only two characteristics showed statistical significance; they were residency in the rural areas (AOR 1.6 [95% CI 1.1–2.4]; *p* = 0.046), and being illiterate (AOR 2.1 [95% CI 1.1–4.0]; *p* = 0.037).

### Life style, family support and physical conditions

The associations between geriatric depression and life style, family support and physical well-being variables have been presented in Table 4.

In the bivariate analyses, the elderly who reported not to be given enough time by their families (OR 3.0 (2.0–4.5); *p* < 0.001), and those who perceived that they received no respect (OR 4.1 [95% CI 1.9–8.7]; *p* < 0.001), and those experiencing verbal and/or physical abuse by their family (OR 3.8 [95% CI 2.2–6.6]; *p* < 0.001) were found to have geriatric depression more frequently.

However, only those elderly who were not given enough time by their families (AOR 1.8 [95% CI 1.1–2.9]; *p* = 0.012) and those who reported verbal and/or physical abuse by their families (AOR 2.6 [95% CI 1.4–4.8]; *p* = 0.002) came out as statistically significant in the multivariate analyses.

Both the bivariate and multivariate analyses showed statistically significant associations between geriatric depression and the presence of any of the chronic physical health problems, and also, between geriatric depression and the inability to move out of the home (limited mobility).

## Discussion

To our knowledge, this is the first community-based door-to-door survey that estimates the prevalence and explores the associations of depression in the elderly population in Nepal. The Kavre district is fairly representative of Nepal as a whole. Depression was assessed using the GDS-15, a validated inventory in Nepali with the cut-off point at scores 5/6.

We found more than half of the elderly population in the Kavre district to suffer from some degree of depression; age and gender standardized prevalence was 53.1%. The risk of having geriatric depression was found to be higher among those residing in the rural areas, and among those who were illiterate. The odds of having a geriatric depression were also higher among those who reported not to be given enough time by their families and among those who reported verbal and/or physical abuse by their families. Furthermore, geriatric depression was more likely to be present among those elderly who had limited mobility and among those who suffered from any of the chronic physical illnesses typical of the elderly.

The prevalence of geriatric depression was slightly lower than what has been found in earlier community-based studies in Nepal [22, 23]. In the past studies, another cut-off point [4/5] was used for the same screening instrument [GDS-15]. The most noticeable finding is that the prevalence of geriatric depression in our study is considerably higher than what has been found in population-based studies from various other Asian countries; 45% in Bangladesh [30]; 30.8% in China [31], 33.8% in Indonesia [32], 33.3% in Japan [33], 39.7% in Korea [34], 48.3% in Laos [35], 22.9% in Myanmar [36], 40.6% in Pakistan [29], 27.8% in Sri Lanka [37], 22.2% in Singapore [38], 27.5% in Taiwan [39], 27.5% in Thailand [40], and 17.2% in Vietnam [33]. It is noteworthy that depression among adults (18–65 years) in a recently conducted nationwide study population in Nepal was also found to be higher than the global mean [41].

In our view, cross-country comparisons should be interpreted with some caution due to methodological differences [11], differences in the instruments and differences in the cut-off points. There are also differences in the age ranges and the socio-cultural settings in which the studies were conducted.

We noticed that most of the studies were carried out in countries relatively more developed than Nepal. Nepal is the country where the primary health care delivery system is largely under-resourced; there is still a lack of trained personnel related to mental health in the primary care settings [42]. In the LAMI countries in general, the majority of the cases of mental illness are untreated because of shortage of mental health professionals [43]. In Nepal, that could be one of the most relevant factors for explaining the higher prevalence of geriatric depression.

We find it most fruitful to compare our findings with Asian studies using the same scale (GDS-15) and the same cut-off point (5/6), and in fairly similar community settings. Even so, the prevalence in Nepal appears to be higher than elsewhere. A similar prevalence figure (48.3%) of geriatric depression, using the same screening instrument at the same cut-off point, was found in Laos [35], a country with economic conditions comparable to those of Nepal.

Socio-cultural factors may affect mental health problems in the underdeveloped societies. Health care practices are largely related to the social norms and values among the community members [11]. In countries like Nepal, there is still a widespread belief that disclosure of mental illness is an embarrassment and may lead to discrimination [41]. The stigma associated with mental illness may contribute towards underreporting as opposed to physical symptoms [44]. Using the same cut-off (5/6) of GDS-15, an almost similar prevalence of geriatric depression (47.0%) was reported from India [28], a country with more or less similar socio-cultural attributes as Nepal.

The literature is conflicting with regard to the age and gender associations of geriatric depression [22, 23, 29, 37]. Though the prevalence among females was higher than for the males, the difference was not found to be statistically significant. The higher prevalence among those above 75 years seen in the bivariate analyses did not survive the multivariate analysis.

In line with other studies [22, 37], we found higher prevalence of depression among the illiterate elderly. Probably illiteracy limits the opportunities to engage in better works or employment. Therefore, those who are elderly and illiterate are more likely to be lower in socio-economic status and financially more insecure [37] in the later stages of life. This may lead to prolonged stress, negative perception of social support, and a lack of control over their social environment. Together, these factors may contribute towards depression [45].

There is a limited literature exploring the associations between geriatric depression and residence in rural areas. In contrast to the findings in Sri Lanka [37], we found the prevalence of geriatric depression to be higher among the elderly residing in the rural areas. The probable explanation could be due to the high level of migration in Nepal of the younger bracket of the population to urban or other countries for employment. Ultimately, the lonely elderly are likely to be home with fewer persons to care for them.

Absence of family support is strongly associated with geriatric depression with a probable bidirectional interaction [46, 47]. The reduced presence of close family members or low social interactions are both recognized as important predictors of depression among the elderly [11, 47, 48]. Consistent with a previous study [22], our study revealed that those elderly who were not given enough time and those reporting physical and/or verbal abuse by their family were more prone to having geriatric depression. The elderly in Nepal rarely live alone, and they rather want to live with their family and there seek love, affection, care and protection. Due to the rapid urbanization and acculturation in the country, the extended family system has been subject to changes from the joint to the nuclear family. The traditional values towards the elderly have also partly been erased; the elderly are more often left alone, neglected, abused and regarded as a burden to the family in contemporary Nepal [49].

The higher odds of having geriatric depression among the participants with one or more chronic physical disease and/or the inability to go out of their homes due to poor mobility have consistently been reported in many studies [22, 50, 51]. Though the relations between chronic physical health problems and geriatric depression could be bidirectional, we believe that long standing physical health problems among the elderly not only imposes a financial burden but also causes functional limitations related to mobility, physical activity in the day-to-day activities. This is likely to undermine the social life of the elderly. Moreover, poor mobility makes the elderly more dependent on others’ presence and involves a loss of autonomy. These problems and implications may negatively affect the psychological well-being of the elderly, and in some cases, this may ultimately result in geriatric depression.

### Strengths and limitations

The very high participation rate (95.4%) within the randomly drawn sample, involving random selection of the rural and urban clusters reduced the chances of participation bias. The careful face-to-face interviews ensured hardly any missing data. Support of the FCHVs during the household survey ensured acceptance in the community. The selected sample was found to be more or less representative of the population of Kavre district, which again is fairly representative of the nation as a whole. We used a standardized and validated instrument for Nepal; it had been used and validated in many other Asian countries, but so far, not in Nepal before this study. These are some strengths of the study.

Of limitations, we want to point out that this is a cross sectional study. We cannot establish causal relationships. The presence of chronic physical health problems was based on self-reports and over- or underreporting may have happened. To minimize this source of error, the trained nurses who served as field interviewers in the data collection, also cross-checked the medical reports or medication in the homes, if any. Using many dichotomous variables for studying the associations may have compromised some of our findings. Due to resource limitations, we were unable to use structured interviews to establish the psychiatric diagnosis of the participants.

## Conclusions

Geriatric depression is highly prevalent in Kavre, Nepal. This mental health condition is associated with socio-demographic factors such as residing in rural areas and illiteracy, low family support in terms of little time given to the elderly and/or exposure to physical and/or verbal abuse, but also the absence of physical well-being. Some of the depressed elderly were unable to go out of their homes, and some suffered from at least one chronic physical health condition. From a public health perspective, there are urgent needs for clearer health-care priorities for the elderly in Nepal. The findings of this study may be used to inform anyone connected to the health care system in the country, and in particular the policy makers for the development of relevant public health and social programs for the prevention and control of depression among the elderly.

## Additional file

Additional file 1: English version of the questionnaire. English version of the questionnaire administered by the field interviewers to the study participants. (PDF 213 kb)

## Data Availability

Personal identification details of the participants were separated from the completed questionnaires and stored in a locked room at the Kathmandu University School of Medical Sciences (KUSMS). No information related to identifiable individuals was disseminated beyond those researchers immediately involved in the study. The data sets used and analyzed in this study are available from the corresponding author on reasonable request.

## Abbreviations

AOR: Adjusted odds ratio
BP: Blood pressure
CBS: Central Bureau of Statistics
CI: Confidence interval
COPD: Chronic obstructive pulmonary diseases
FCHV: Female community health volunteer
GBD: The Global Burden of Disease
GDS: Geriatric Depression Scale
ICD: International Classification of Disease
IRCKUSMS: Institutional Review Committee of Kathmandu University School of Medical Sciences
LAMI: Low-and middle-income
OPD: Out patients department
OR: Odds ratio
QoL: Quality of life
SD: Standard deviation
Se: Sensitivity
SEAR: South-East Asia Region
Sp: Specificity
UGC: University Grants Commission
YLD: Years of life lost to disability
