# Supplementary material for: Prevalence of geriatric depression in the Kavre district, Nepal: Findings from a cross sectional community survey
Source: BMC Psychiatry. 2019 Sep 3;19:271. doi: 10.1186/s12888-019-2258-5 (PMC6724336; doi:10.1186/s12888-019-2258-5)
Supplement: Supplementary file 1 — English version of the questionnaire. English version of the questionnaire administered by the field interviewers to the study participants. (PDF 213 kb) [file 12888_2019_2258_MOESM1_ESM.pdf]

Kathmandu University, School of Medical Sciences, Dhulikhe Hospital, Dhulikhel, Kavre
2019

Nepali Version Geriatric Depression Survey Instrument
For
Prevalence, associations, and family caregiver burden of geriatric depression in Kavre District: A community-based cross-sectional educational project

Nepali version

For administration by trained nurses or health workers

Information need to be entered by interviewer	
Form No (filled in center level)	…………………………….
Place of residency	Urban/rural municipality
Code No of Urban/Rural municipality	………………………
Household number	………………………….
Date of data collection	DD/MM/YYYY
Name of data interviewer	………………………….
Signature of interviewer (on completion)	………………………….

Demographic Information		
**Questions**		**Response**
1.	Sex (*record male female as observed)*	• Male<br>• Female
2	What is your date of birth?	DD MM YYYY
3	How old are you? (completed year)	………………………
4	Residence	• Urban municipality<br>• Rural municipality
5	In total, how many years have you spent in school or full time study (excluding pre-school)	…………………………..
6	What is your educational level	• Illiterate<br>• No formal schooling<br>• primary level<br>• Primary level completed<br>• Secondary level completed<br>• Higher secondary level completed<br>• Bachelor degree completed<br>• Master degree or above level completed
7	What is your marital status?	• Never married<br>• Married living with spouse<br>• Separated<br>• Divorced<br>• Widowed<br>• Cohabitating<br>• Refused
8	Which of the following best describes your main work status over the past 12 months?	• Government employee<br>• Non-government employee<br>• Self-employee<br>• Non-paid<br>• Homemaker<br>• Retired<br>• Unemployed (able to work)<br>• Unemployed (unable to work)
**Smoking**		

Next questions about ask questions on smoking		
9	Do you currently smoke any tobacco products such as cigarettes; cigar pipes bidis, hukahs or tamakhus?	• Yes<br>• No **(if no, please go question no 12)**
10	Do you currently smoke tobacco products daily?	• Yes<br>• No
11	How old you when you first started smoking?	....
12	In the past, did you smoke any tobacco products?	• Yes<br>• No **(if no, please go question no 12)**
13	In the past, did you smoke daily?	• Yes<br>• No
14	How old were you when you stopped smoking?	....
**Alcohol consumption**		
Next questions ask about alcohol consumption		
15	Have you consumed an alcoholic drink such as beer, wine, whisky, spirits, fermented cider (jaad, raksi, tumba) within the past 12 months?	• Yes<br>• No
16	During the past 12 months, how frequently have you had at least one alcoholic drink?	• Daily<br>• Few days of a weeks<br>• Weekly<br>• Monthly<br>• Occasionally
17	Have you ever consumed an alcoholic drink?	• Yes<br>• No
**Social supports**		
Next questions ask about social supports		
18	With whom, are you staying at home?	• Alone<br>• With spouse<br>• With other family members
19	Are you head of household?	• Yes<br>• No
20	Do you usually get time from your family member?	• Yes, enough time<br>• Yes, but little bit<br>• Not at all
21	How do family decide about family concern issues	• Yourself<br>• Consulting with you<br>• Without consulting with you

22	How do you manage your financial requirement?	• From your work<br>• Retirement found<br>• Bank deposit<br>• Family support<br>• Not at all<br>• Others (specify….)
23	How do you manage your daily activities such as cooking food, washing cloth?	• Yourself<br>• Helpers (other than family member)<br>• Other family member
24	Can you go outside from your house for your social activities (meeting with your friends, relatives, shopping, party, religious etc)	• Yes, independently myself<br>• Yes independently with assistant of device<br>• Yes but support of family members<br>• No, I cannot go
25	Do you feel respect from your family members and other relatives	• Yes<br>• No
26	Have you experiences of verbal abuse from your family member in last 12 months?	• Usually<br>• Sometimes<br>• Never
27	Have you experiences of physical assault from your family member in last 12 months?	• Usually<br>• Sometimes<br>• Never
**Physical chronic health problems**		
Self-reported physical health problems (if possible look the medical records and medicine they used for treatment)		
28	Diabetes<br>Chronic obstructive pulmonary diseases (COPD)<br>Heart diseases<br>Cancer<br>Stroke<br>Others (specify……………………)	• Yes No Medications<br>• Yes No Medications<br><br>• Yes No Medications<br>• Yes No Medications<br>• Yes No Treatment<br>• Yes No Medications
**Quality of life questions (WHOQoL-8)**<br><br>This set of eight questions, developed by the World Health Organization to measure quality of life.<br><br>The questions ask how you feel about your quality of life, health or other areas of your life. Each question has five response options. **Please choose the answer that appears most appropriate by**		

circling the number in the appropriate column. If you are unsure about which response to give to a question, the first response you think of is often the best one.

Please keep in mind your standards, hopes, pleasures and concerns. We ask that you think about your life **in the last 4 weeks.**

		very poor	poor	neither poor nor good	good	very good
29	How would you rate your quality of life?	1	2	3	4	5
		**Very dissatisfied**	**dissatisfied**	**neither satisfied nor dissatisfied**	**satisfied**	**very satisfied**
30	How satisfied are you with your health?	1	2	3	4	5
31	How satisfied are you with your ability to perform your daily living activities?	1	2	3	4	5
32	How satisfied are you with yourself?	1	2	3	4	5
33	How satisfied are you with your personal relationships?	1	2	3	4	5
34	How satisfied are you with the conditions of your living place?	1	2	3	4	5
		**not at all**	**a little**	**moderately**	**mostly**	**completely**
35	Do you have enough energy for everyday life?	1	2	3	4	5
36	Have you enough money to meet your needs?	1	2	3	4	5

Geriatric Depression Scale (short form)
Instructions: These 15 questions ask about depression. Please give the answer that best describes how you felt over the past week.

37	Are you basically satisfied with your life?	Yes	No
38	Have you dropped many of your activities and interest?	Yes	No
39	Do you feel life is empty?	Yes	No
40	Do you often get bored?	Yes	No
41	Are you in good spirits most of time?	Yes	No

42	Are you afraid that something bad is going to happen to you?	Yes	No
43	Do you feel happy most of time?	Yes	No
44	Do you often feel helpless?	Yes	No
45	Do you prefer to stay at home, rather than going out and doing things?	Yes	No
46	Do you feel that you have more problems with memory than most?	Yes	No
47	Do you think it is wonderful to be alive now?	Yes	No
48	Do you feel worthless the way you are now?	Yes	No
49	Do you feel full of energy?	Yes	No
50	Do you feel that your situation is hopeless?	Yes	No
51	Do you think that most people are better off than you are?	Yes	No
**General fitness questions**<br>These measurements will give an indication of your level of fitness.			
52	**Weight?**<br>(please enter weight in ordinary clothing, without shoes, in kilograms)	________ kg	
53	**Height?**<br>(please enter height, without shoes, in centimeters)	________ cm	
54	**Waist measurement?**<br>(please measure at the level of the umbilicus (navel) and enter the measurement in centimeters)	_______ cm	
55	**Blood pressure?**<br>**Instructions:**<br>Please take two measurements following these instructions:<br>• from the right arm (unless this is not possible for any reason)<br>• with the arm elevated so that the cuff is at heart level<br>• with the subject sitting with feet on floor<br>• after 5 minutes' rest	First:<br>______/______<br>mm Hg<br><br>Second:<br><br>______/______<br>mm Hg	
56	Are you taking anti-hypertensive drugs?	Yes<br>No	
**The questionnaire is now complete. Thank you very much for your time.**			
